# Regular Nanowire Formation on Fe-Based Metal Glass by Manipulation of Surface Waves

**DOI:** 10.3390/nano11092389

**Published:** 2021-09-14

**Authors:** Zhen Zhao, Chaoqun Xia, Jianjun Yang

**Affiliations:** 1 GPL Photonics Laboratory, State Key Laboratory of Applied Optics, Changchun Institute of Optics, Fine Mechanics and Physics, Chinese Academy of Sciences, Changchun 130033, China; zhaozhen17@mails.ucas.ac.cn; 2 Center of Materials Science and Optoelectronics Engineering, University of Chinese Academy of Sciences, Beijing 100049, China; 3 School of Materials Science and Engineering, Hebei University of Technology, Tianjin 300130, China; chaoqunxia@hebut.edu.cn

**Keywords:** magnetic surface wave, femtosecond laser, metallic glass, nanostructures

## Abstract

We report the formation of a sole long nanowire structure and the regular nanowire arrays inside a groove on the surface of Fe-based metallic glass upon irradiation of two temporally delayed femtosecond lasers with the identical linear polarization parallel and perpendicular to the groove, respectively. The regular structure formation can be well observed within the delay time of 20 ps for a given total laser fluence of *F* = 30 mJ/cm^2^ and within a total laser fluence range of *F* = 30–42 mJ/cm^2^ for a given delay time of 5 ps. The structural features, including the unit width and distribution period, are measured on a one-hundred nanometer scale, much less than the incident laser wavelength of 800 nm. The degree of structure regularity sharply contrasts with traditional observations. To comprehensively understand such phenomena, we propose a new physical model by considering the spin angular momentum of surface plasmon and its enhanced inhomogeneous magnetization for the ferromagnetic metal. Therefore, an intensive TE polarized magnetic surface wave is excited to result in the nanometer-scaled energy fringes and the ablative troughs. The theory is further verified by the observation of nanowire structure disappearance at the larger time delays of two laser pulses.

## 1. Introduction

The creation of micro and nanoscale structures is known as a cornerstone for the development of modern photonics because it possesses capabilities in manipulating optical properties of materials for the improved performance of devices [1,2]. Currently, several top-down and bottom-up fabrication methods have already been established for this purpose, which often, however, face major hurdles of lack reproducibility, high cost, and rigorous conditions [3,4]. On the contrary, the recent advancements of femtosecond laser applications have clearly demonstrated an alternative potential for the large-area production of the periodic structures in subwavelength or even nanometer scales with a convenient mask-free way, which are verified on a variety of materials, including metals [5,6,7,8,9,10,11,12,13,14], semiconductors [15,16,17], and dielectrics [18,19,20,21]. Numerous studies have suggested that the formation of such periodic structures depends on the laser parameters, material properties, and ambient conditions.

Especially for the solid metallic materials, the laser-induced periodic surface structures (LIPSSs) are usually categorized into the low-spatial-frequency (LSF) and the high-spatial-frequency (HSF) types [5,6,7,8,9,10,11,12,13,14,15,16,17,18,19,20,21]. In the former case, the structures often exhibit a spatial period (*Λ*) close to the incident laser wavelength (*λ*), i.e., (*λ*/2 < *Λ* < *λ*), associated with orientation perpendicular to the direction of the light polarization. The underlying mechanisms are basically attributed to interference between the incident laser and the excited TM-mode surface plasmon polaritons (SPP) [7,8,9,10,11,12,13,14,15,16]; whereas in the latter case, the structures possess the spatial period much less than the incident laser wavelength, i.e., *Λ* < *λ*/2, with orientation either parallel or perpendicular to the light polarization [21,22,23,24]. In spite of the Sipe theory explanation [25], their physical origins are still under debate in the literature, including the interpretations based on the framework of electromagnetic theory [26] and self-organization [27]. Recently, Colombier et al. tried to understand the HSF structure formation with a hydrodynamic theory, wherein the surface roughness can effectively transform the laser energy into the temperature gradients for the hydrothermal wave generation and the material redistribution [28]. However, their explanation for the effect of the electron-phonon coupling on the structure formation is contradictory to the experimental observations by Ionin et al. [29]. Moreover, according to this theory, the degree of the structure’s regular arrangement is independent of the surroundings. Another common problem, especially for the HSF structures, is showing wavy, bending, and fragmentation appearances, which poses a serious challenge to the surface photonic device applications. Although Obara et al. proposed that the spatial arrangement of laser-induced structures can be controlled by the existing nano-geometries via the plasmonic and Mie scattering [30], the corresponding results for the HSF structures are rarely reported.

In this letter, we investigate an intriguing formation of the nanowire structures within a pre-fabricated groove on Fe-based metallic glass, using two time-delayed femtosecond laser beams linearly polarized in the same direction. It is found that the laser-induced structure profiles can present either a sole long nanowire or the regular nanowire arrays depending on the laser polarization with respect to the groove direction. In particular, the underlying mechanisms are then discussed with the help of considering an inhomogeneous magnetization effect induced by the surface plasmon on the ferromagnetic metal, which consequently generates the magnetic surface wave to result in the anomalous arrangement of the nanometer-scaled structures. The further experimental results can support the theory.

## 2. Materials and Methods

**Figure 1:**
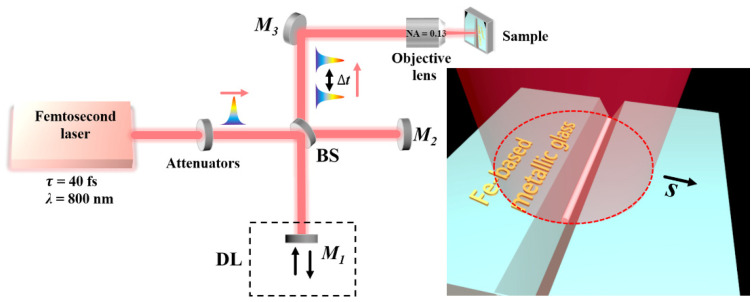
Schematic diagram of the experimental setup for the sole long nanowire structure formation inside the pre-scratched groove on the surface of Fe-based metallic glass, upon irradiation of the temporally delayed femtosecond laser pulses. *τ*: pulse width; *λ*: central wavelength of laser; BS: beam splitter; DL: delay line; Δ*t*: delay time; M: reflector; *S*: direction of sample translation.

The experimental schematic design is shown in Figure 1, wherein a commercial Ti: sapphire femtosecond laser amplifier (Spitfire Ace, Spectra Physics, Santa Clara, CA, USA) delivers the linearly polarized pulse trains with the central wavelength of 800 nm and the time duration of 40 fs. Operation at a repetition rate of 1 kHz makes two successive laser pulses temporally separated by one millisecond, which makes the direct coupling between their interaction with the material become negligible. To explore the effects of dual correlated laser-material interaction dynamics, the generation of laser pulses with a picosecond temporal delay is required. Thus, we established a Michelson interferometer-like configuration to divide a single laser pulse out of the laser system into two identical sub-pulses propagating in different arms. The optical length of one arm can be adjusted via a delay-line to precisely change the traveling time of the laser sub-pulses. Afterward, the spatial separation of two laser sub-pulses was modulated by a beam splitter into an overlapping collinear propagation, and then they were focused by an objective lens (Nikon, Tokyo, Japan, 4×, NA = 0.13, NA: numerical aperture) onto the sample surface at normal incidence. Before dividing the laser pulse, the neutral density attenuators were inserted to adjust the laser energy. Of course, irradiation of a millisecond time-delayed femtosecond laser pulse onto the target can be simply obtained by blocking one of two optical arms.

**Figure 2:**
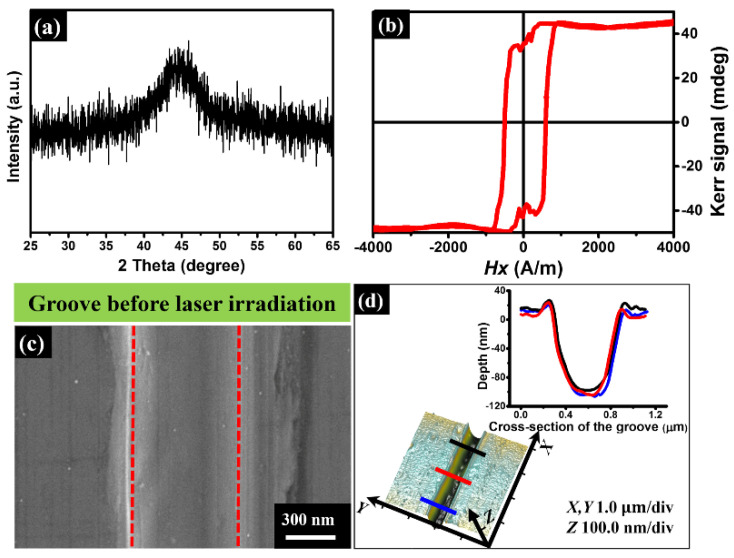
(**a**,**b**) Measured XRD curve and hysteresis loop for the sample of Fe-based metallic glass before the femtosecond laser irradiation. (**c**,**d**) SEM and AFM images of the mechanically scratched groove on the surface of Fe-based metallic glass. The two red dashed lines identify the boundaries of the groove. Upper-right inset in (**d**) shows the measured cross-section profiles at three different positions marked by the red, black, and blue lines.

In the experiment, a 40 μm-thick foil of Fe-based metallic glass (Fe_85_Si_9_B_6_) was selected as a sample material because of its amorphous state for the superior physical and chemical properties, including superplasticity, wear and corrosion resistance, and excellent soft magnetic behavior [31,32,33]. Before irradiation of the laser pulses, both the amorphous property and the magnetic response of our sample were characterized by an X-ray diffractometer (Rigaku, Tokyo, Japan) and a vibrating sample magnetometer (Lakeshore, Westerville, OH, USA), respectively, as shown in Figure 2a,b. Clearly, the measurement result of X-ray diffraction (XRD) reveals only one broad scattering peak around 2*θ* = 40–50° for Cu Kα radiation of 1.541 Å, being a typical feature of the Fe-based amorphous alloy. The hysteresis loop measurement was obtained at 300 K with an applied magnetic field of 4000 A/m, convincingly showing the soft magnetic properties of the sample material.

For the purpose of investigating the influential factors for the laser-induced structures, a shallow groove was mechanically scratched on the sample surface, whose morphology is characterized by both a scanning electron microscope (SEM, Phenom, Eindhoven, The Netherlands) and an atomic force microscope (AFM, Bruker, Billerica, MA, USA), as shown in Figure 2c,d. The cross-section measurement of the groove appears to show the nearly U-shaped geometry, with the upper opening width and the longitudinal depth of 600 ± 9 nm and 110 ± 9 nm, respectively. The sample material was fixed on a computer-controlled three-axis translation stage. In order to avoid the strong ablation damages, the sample surface was placed at the position of 600 μm before the laser focus and the estimated beam spot on the sample surface of approximately *w*_z_ = 78 μm in radius. All the experiments were carried out in an ambient air environment through scanning of the sample at the speed of *v* = 0.3 mm/s, resulting in *N* = 520 laser pulses partially overlapped within one spot area. The peak fluence of the laser pulse was given by *F* = 2*E*_0_/π*w_z_*^2^, where *E*_0_ represents the pulse energy on the sample surface. Moreover, the measured optical index of the material via a commercial ellipsometer (Semilab, Budapest, Hungary) is given by *n* = 3.06 + *i* 3.95 for the laser wavelength of *λ* = 800 nm. 

## 3. Results and Discussions

**Figure 3:**
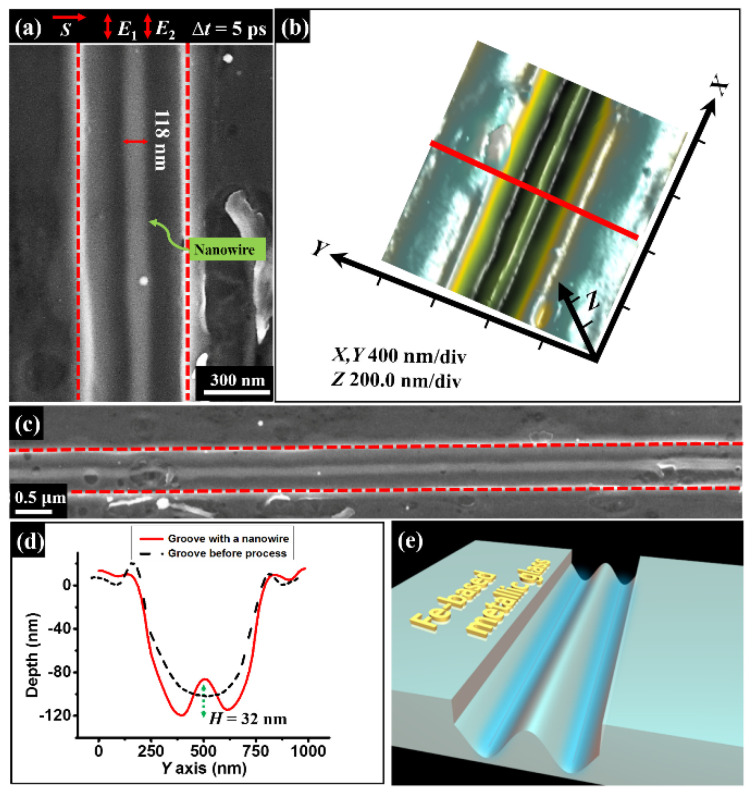
Observed formation of the sole long nanowire structure within the groove irradiated by double femtosecond lasers with the time delay of ∆*t* = 5 ps, where the total energy fluence of two lasers is *F* = 30 mJ/cm^2^, and the scanning speed is *v* = 0.3 mm/s. (**a**,**b**) SEM image and three-dimensional AFM measurement of the sole nanowire structure inside the groove. Here *E*_1_ and *E*_2_ with the red double arrows represent the direction of the laser polarization, and *S* with the red single arrow for the sample translation direction. (**c**) SEM picture for the 10 μm length of the sole nanowire structure. (**d**) Measured cross-section curves for the groove before (black line) and after (red line) the femtosecond laser irradiation. (**e**) A diagram of the nanowire structure formation inside the groove, where the blue color lines represent the interfering intensity fringes between the femtosecond laser and TE polarized magnetic surface wave inside the groove.

Figure 3 shows the experimental results when the femtosecond laser irradiation is scanned across the groove, in which double laser sub-pulses are temporally delayed by ∆*t* = 5 ps with the same energy fluence of *F*_1_ = *F*_2_ = 15 mJ/cm^2^ and the identical linear polarization (*E*_1_ and *E*_2_) parallel to the groove direction. From the SEM image, we can surprisingly find that a long uniform nanowire structure is solely formed on the bottom of the groove, with a measured width of about 118 nm. In other words, such a structure formation is parallel to the directions of both the laser polarization and the groove, with the geometric dimension much smaller than the incident laser wavelength. Moreover, such a nanowire structure formation can be continuously extended to a long distance for the total laser energy fluence ranging from *F* = 30 mJ/cm^2^ to 42 mJ/cm^2^, and the experimental observation in Figure 3c is only taken for example. The AFM measurement result revealed that the cross-section of the single nanowire structure presents a cone-like profile, associated with a modulation height of approximately *H* = 32 nm, as shown in Figure 3d. Noticeably, in sharp contrast to the AFM image of the groove before the laser irradiation, the single nanowire formation within the groove originated from the laser irradiation rather than the scratching process.

**Figure 4:**
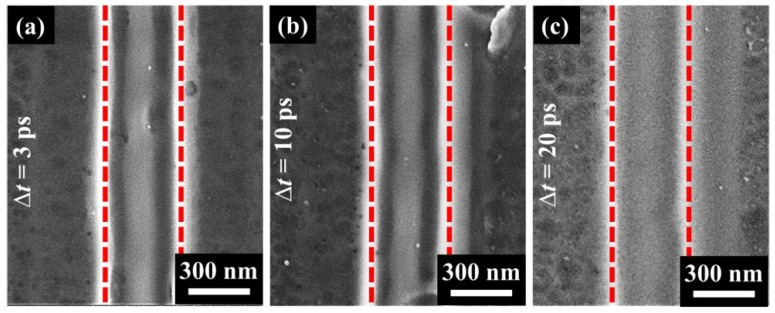
Formation of the sole long nanowire structure inside the groove with varying time delay of laser sub-pulses. (**a**) Δ*t* = 3 ps, (**b**) Δ*t* = 10 ps, (**c**) Δ*t* = 20 ps. The total incident laser energy fluence is *F* = 30 mJ/cm^2^, and the scanning speed is *v* = 0.3 mm/s.

Figure 4 shows the experimentally observed effects of the time delays (Δ*t*) between double laser sub-pulses on the nanowire structure formation inside the groove, wherein the employed other laser parameters are identical to those in Figure 3. Interestingly, for the time delays, no more than Δ*t* = 10 ps, the formation of the sole long nanowire structure can be observed clearly. When the time delay was increased to Δ*t* = 20 ps and even larger, however, it is hard to distinguish the nanowire formation. As a matter of fact, a comparative experiment was also conducted by using the single-beam femtosecond laser irradiation at a 1 kHz repetition rate within a laser energy fluence range of *F* = 30–42 mJ/cm^2^, but the sole long nanowire structure was no longer formed inside the groove.

**Figure 5:**
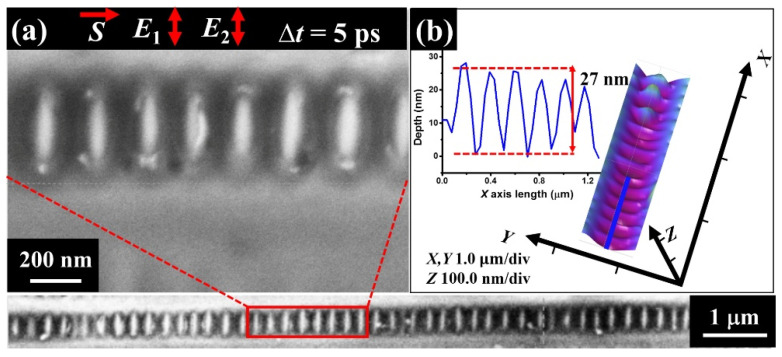
(**a**,**b**) SEM and AFM images of the laser-induced regular periodic arrays of short-lengthed nanowire structures inside the groove on Fe-based metallic glass. The left inset in (**b**) shows the measured cross-section curve for a small portion (blue line) of the nanostructure arrays in the groove.

In order to verify the dependence of the structure formation on the laser polarization, a further experiment was carried out by only rotating the groove direction at 90 degrees while keeping the identical laser scanning, during which the laser polarization of both beams became perpendicular to the groove direction; the corresponding results are shown in Figure 5. Clearly, from the SEM image, we can unprecedentedly find the periodic arrays of short-lengthed nanowire structures formed in the groove, whose orientation becomes perpendicular to the groove direction but still keeps parallel to the direction of the incident laser polarization. Remarkably, such periodic structure arrays can be evidenced distinctly within a long range of the groove, illuminated by the femtosecond lasers. The measured width of each unit approximates 100 nm, associated with the arrangement period of about *Λ* = 192 nm. All these geometric features are much smaller than the incident laser wavelength, entering into the deep subwavelength scales. Undoubtedly, neither the structure period nor spatial orientation can be explained by the traditional theory of TM-polarized SPP excitation. Moreover, the corresponding AFM results reveal that the observed nanowire arrays are, in fact, the narrow ridges of the periodic ablation troughs within the groove, and the measured cross-section curve presents the modulation depth of about *H* = 27 nm. 

**Figure 6:**
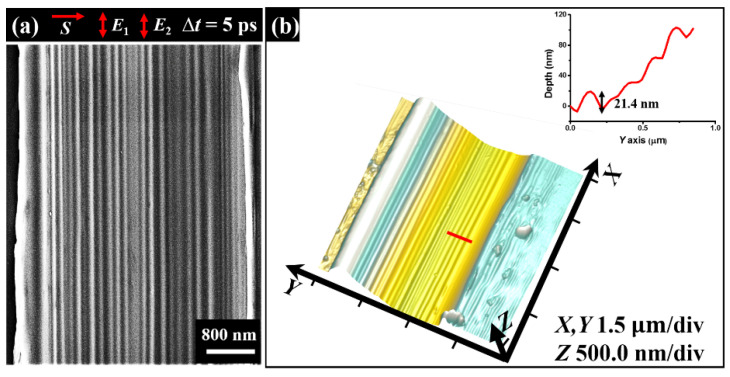
Observed formation of the multiple regular nanowire structures within a wide groove of 3.6 μm under irradiation of femtosecond laser pulses. (**a**,**b**) SEM and AFM images, respectively. Upper-right inset in (**b**) reveals the measured height of the nanowire structures marked by the red line in AFM image.

In order to verify the spatial restriction of the groove width for the formation of the sole long nanowire structure, we employed a wide scratched groove with the opening width of 3.6 μm to repeat the above experiment; the corresponding results are shown in Figure 6. In this case, there are multiple nanowires regularly formed on the surface of the groove bottom, with orientations parallel to the direction of the laser polarization. Except for the increasing number of nanowire growth, all the structure features, including the modulation height of 21.4 nm and structure period of 185 nm, are nearly consistent with the sole nanowire structure. It should be noticed that our observation of the highly regular HSF structures oriented parallel to the laser polarization cannot be well understood by the hydrodynamic theory because the material of Fe-based metallic glass tends to have a large electron-phonon coupling constant of 1.7 × 10^18^ W/m^3^/K [34].

**Figure 7:**
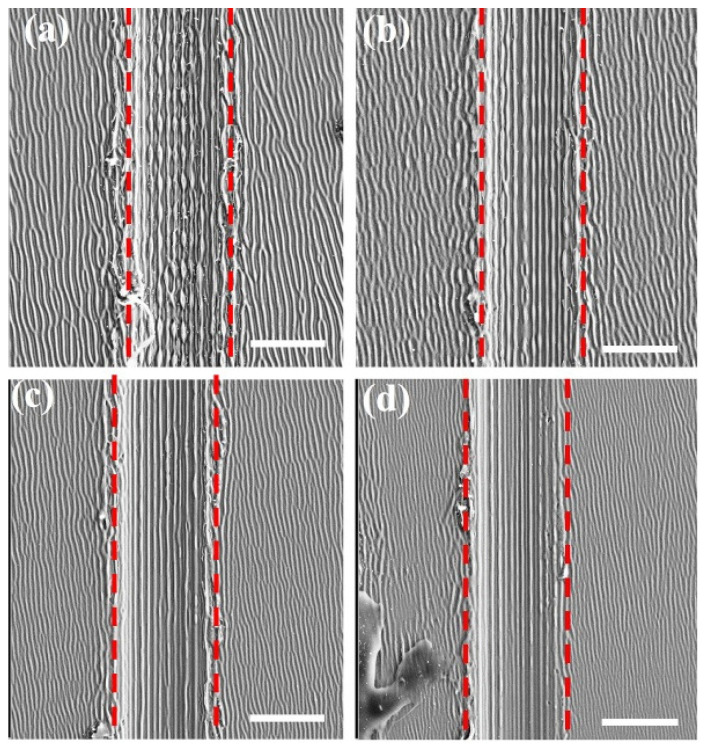
Observed evolution of the laser-induced structure morphology with different total incident laser fluences. (**a**) 48 mJ/cm^2^, (**b**) 46 mJ/cm^2^, (**c**) 42 mJ/cm^2^, and (**d**) 40 mJ/cm^2^. Scale bar: 2 μm.

Under such circumstances, the evolution of surface morphology inside the groove, with different total laser fluences, is shown in Figure 7. From the SEM image in Figure 7a, we find that when the total laser fluence is *F* = 48 mJ/cm^2^, the laser-induced parallel nanowires inside the groove are interrupted by the periodic formation of nano-bumps. Interestingly, with reducing the total laser fluence to *F* = 40 mJ/cm^2^, the periodic distribution of the nano-bumps gradually disappeared, which makes the formation of nanowires smooth. Such formation of the composite structure originates from hydrodynamic instabilities, i.e., during the material cooling process, the surface tension of the liquid column causes hydrodynamic instabilities, whose disturbance makes it break into drops, leading to the periodic distribution of nano-bumps [35]. However, in contrast to the higher laser fluences, the lower laser fluences shrink the cooling time for the melting material so that the nano-bumps cannot be developed before the material re-solidification.

**Figure 8:**
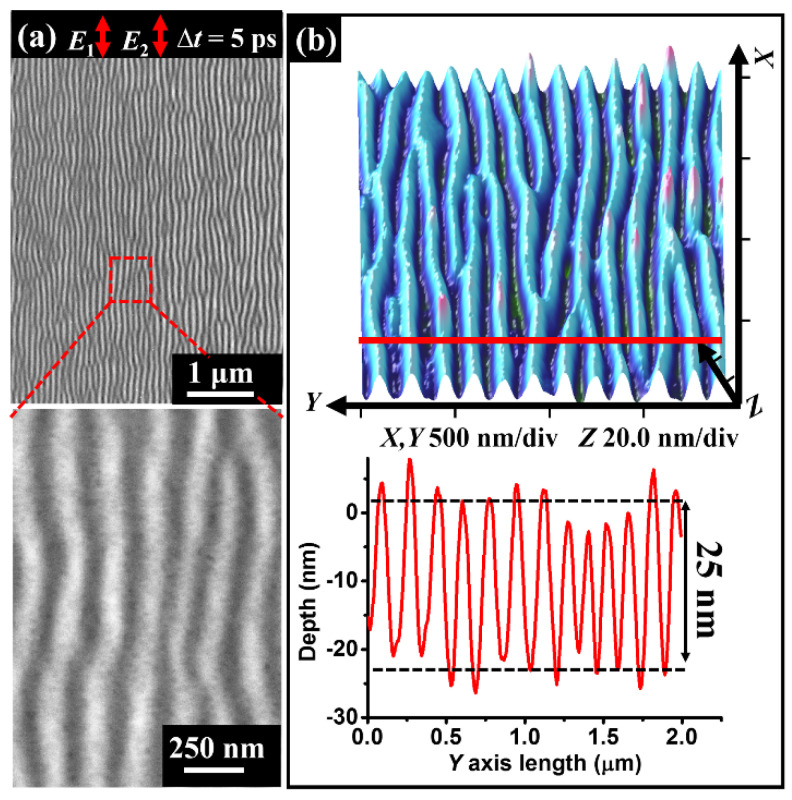
(**a**,**b**) SEM and AFM images of LIPSSs on the flat surface of Fe-based metallic glass. Bottom illustration in (**b**) shows the measured cross-section curve for a small portion (Red line) of the structure formation on material surface.

Apart from the grooved sample surface, the formation of multi-line LIPSSs with orientation parallel to the direction of the laser polarization was also evidenced on the flat surface of the sample material when two time-delayed femtosecond laser pulse trains identical to Figure 3 are adopted except for the incident total energy fluence of *F* = 45 mJ/cm^2^, and the corresponding results are shown in Figure 8. Compared with the regular patterns within the groove, we find here that the spatial distribution of LIPSSs on the flat surface is twisted into the bending, interruption, and bifurcation features. In this case, the measured structure period and modulation depth approximate *Λ* = 166 nm and *H* = 25 nm, respectively. The wavy arrangement of the structures may be due to the stochastic superposition of the surface wave excitation on the rough surface of the material. For the grooved sample surface, the existing groove edges can be considered as the evident scattering sources for the surface wave, and such wave propagation is caused by the coherent superposition of the fields irradiated from these sources so that the spatial alignment of the structure become more regular [36]. That is, the geometric features of the pre-scratched groove are essential to improve the spatial regularity of the nanostructure formation. Moreover, further experiments reveal that the HSF structures can be generated within a laser energy fluence ratio range of 1/3 < *F*_1_/*F*_2_ < 3.

**Figure 9:**
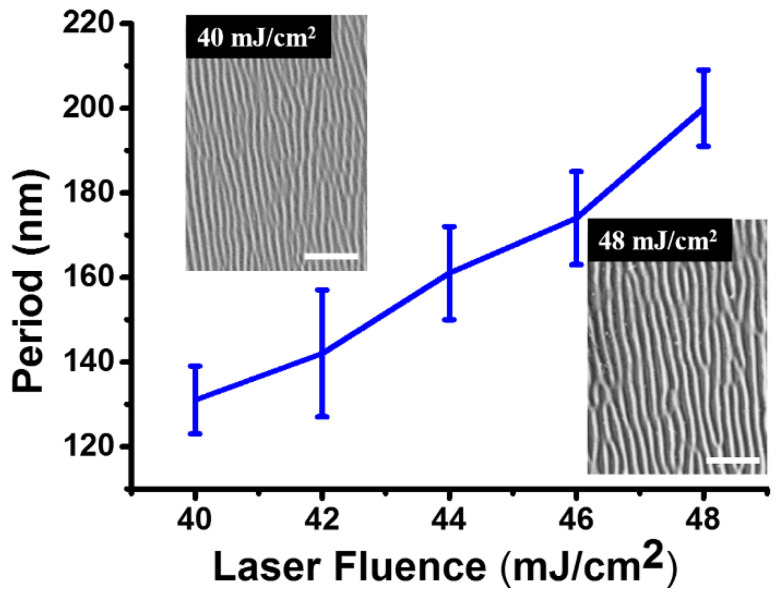
Measured variations of the structure period as a function of the incident laser energy fluence of two beams. Both upper-left and bottom-right insets show the SEM images (scale bar: 1 μm) of the nanostructure under the laser fluences of *F* = 40 mJ/cm^2^ and 48 mJ/cm^2^, respectively.

Moreover, we successfully achieved the dependence of the structure period on the laser fluence in a range of *F* = 40–48 mJ/cm^2^, based on a large number of experimental measurements (Figure 9); wherein, the employed laser parameters are the same as Figure 3, and each value is obtained from the average measurement of 20 nanostructures. Clearly, by increasing the laser energy fluence, the structure period exhibits larger variations. More specifically, when the incident laser fluence was increased from *F* = 40 mJ/cm^2^ to 48 mJ/cm^2^, the period of the structures gradually enlarged from *Λ* = 131 nm to 200 nm. 

Apparently, the formation of the sole long nanowire structure sharply contrasts with the traditional observation of LIPSSs that usually consists of multiple parallel lines. To understand such a curious phenomenon, we should comprehensively understand the physical processes of the interaction between femtosecond laser and Fe-based metallic glass. Usually, the LIPSS formation with orientation perpendicular to the laser polarization has been widely attributed to a collective motion of the conduction electrons on the material surface, or SPP excitation, upon the femtosecond laser irradiation, and the maximum intensity of its interference with the incident laser should exceed the material ablation threshold [29]. In the experiment, our sample material is like other metals to possess free electrons, and thus, is capable of supporting the TM-mode SPP excitation. 

**Figure 10:**
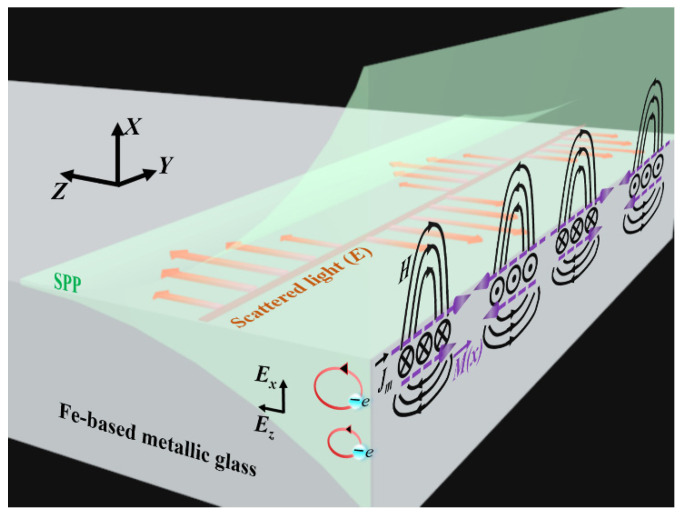
Schematic diagram of the physical processes for the TE-MSW generation on magnetic Fe-based metallic glass surface.

In 2019, Maksimovic et al. reported that the laser-induced periodic microstructures on the Si surface could be controlled by the external applied magnetic field, which was attributed to the modulation of the magnetic field for the density and dynamic of the SPP [37]. More interestingly, recent studies reveal that the TM-SPP wave on the metal surface can lead to another physical effect of spin angular momentum because a rotation of the electric-field vector within the propagation plane is generated by a π/2 phase difference between the two electric components [38]. When taking into account the local motion of electrons, the microscopic orbital motion of electrons in the SPP field is obtained and can produce multiple circulating currents, and hence a transverse magnetization of the metal [39]. In other words, the SPP field, being a mixed photon-electron excitation, carries a non-zero magnetic moment. The magnetization ($\vec{M}$) can be calculated by the following formula [39]:

(1) $$\vec{M}=g{\left|A\right|}^{2}\frac{-e}{2mc}\frac{2\left(1-\varepsilon \right)\sqrt{-\varepsilon }}{\varepsilon^{2}}exp\left(2k_{2}x\right)\frac{\left(\vec{E_{z}}\times \vec{E_{x}}\right)}{\left|\vec{E_{z}}\times \vec{E_{x}}\right|}$$

where g is Gaussian units (8π*ω*)^−1^, *e* is electron charge, *m* for electron mass, *c* for the light velocity, *ω* for the angular frequency of laser light, *ε* for the material permittivity, *A* for the SPP field amplitude, *k*_2_ for the spatial decaying constant of the SPP field within the material, *x* for the spatial coordinate perpendicular to interface, *E_x_* and *E_z_* for the electric field components of SPP in two directions. The above equation indicates that the effect of SPP excitation on the magnetic system is equivalent to the application of an external magnetic field pulse. Furthermore, owing to the intrinsic properties of the SPP field intensity decaying normal to the surface, the spatially inhomogeneous magnetization is eventually achieved, which subsequently generates the corresponding magnetization electric current by the relationship $\vec{j_{m}}=c\nabla \times \vec{M}$, with flowing in the metal along the SPP propagation, as shown in Figure 10. 

On the other hand, for the scattered light along the direction (of the *Y*-axis) perpendicular to the magnetization current, it possesses the electric field oscillating harmonically in time and space. Therefore, when the SPP excitation is relatively weak, both the magnetization and the induced current on the material surface are physically dominated by the combination of the scattered light and the *E_x_* component of the SPP field, which of course, harmonically oscillate along the *Y*-axis. It is also recognized that the induced surface magnetization current tends to result in a discontinuity of the tangential magnetic field at the metal surface, and then a transverse electric polarized magnetic surface wave (MSW) becomes possible [40]. Furthermore, with the gradual enhancement of the SPP excitation, the predominant role of the scattered light will be submerged, and the TE-MSW cannot be developed. Namely, only in the case of the weak SPP excitation would the free electrons-based TM mode SPP transfer into a TE mode MSW, the predominance of which relies on the material property.

For non-ferromagnetic metals, the inhomogeneous magnetization within the metal is likely to disappear at the sub-picosecond timescale (the lifetime of SPP field) [41], leading to no correlations among the material magnetization by the femtosecond laser irradiation, especially operating at a 1 kHz repetition rate (with a millisecond temporal interval between two neighboring pulses). Accordingly, each process of TE-MSW excitation is physically independent, and their weak intensity is not enough to ablate the material surface. However, for the ferromagnetic materials with hysteresis effect, the inhomogeneous magnetization can be gradually accumulated during multi-pulse femtosecond laser irradiation, even with a rapid attenuation process for each magnetization.

Additionally, the TE-MSW excitation originates from the inhomogeneous magnetization driven by SPP, wherein the demagnetization is also involved due to the heat effect of the laser irradiation. In other words, there is a competition between magnetization and demagnetization during the laser process. For the larger time delays, the demagnetization effect plays the dominant role because of the weaker excitation of TE-MSW, which makes it difficult to form the structure on the sample surface [42]. However, for shorter time delays, the inhomogeneous magnetization on the ferromagnetic material can be accumulated for higher strength during the multi-pulse femtosecond laser irradiation, leading to a stronger generation of TE-MSW to imprint the structures on the sample surface.

Afterward, the optical interference of the incident light with the excited TE-MSW causes periodic fringes, whose intensity can exceed the damage threshold of the material. Furthermore, their spatially localized ablation leads to periodic valleys oriented parallel to the groove direction. According to [43], the propagation vector of the excited TE-MSW can be estimated by:

(2) $$k_{s}=\frac{\omega }{c}\sqrt{\left(\frac{\mu_{2}\varepsilon_{1}-\mu_{1}\varepsilon_{2}}{\mu_{2}-\mu_{1}}\right)\frac{\mu_{1}\mu_{2}}{\mu_{1}+\mu_{2}}}$$

with *μ_i_* and *ε_i_* (*i* = 1, 2) being the real parts of the magnetic permeability and dielectric permittivity of the media, respectively. In our case, the parameters for the surrounding air medium (*i* = 1) and the sample material (*i* = 2) are assumed to be *μ*_1_ = *ε*_1_ = 1.00, *μ*_2_ = 1.20, *ε*_2_ = −6.30, respectively. Thus, the spatial period of the laser-induced structures is given by *Λ_s_* = 2π/*k_s_* = 177 nm. Because the flat region of the groove bottom is only about 250 nm in width, at most, two intensity fringes can be contained inside, and the observation of sole long nanowire structure is, in fact, the in-between ridge material with a bulging profile caused by thermal ablation of the bilateral valleys (Figure 3e). In other words, the narrow width of the groove bottom can act as a spatial gate to select the number of the intensity fringes and their ablation results. On the other hand, for the inner wall surfaces of the U-shaped groove, the laser irradiation would likely have the near grazing incidence angles resulting in much less effective laser energy, and thus, no periodic ablation valleys.

It is worth mentioning that during our experiments, no plasmonic field can be scattered from the groove edges because the linear polarization of the incident femtosecond lasers are parallel to the groove orientation [36], and thus, there is no need to consider its physical influence on the single nanowire formation. In practice, such a sole long nanowire structure in the groove can only take place within a range of the proper laser energy fluences and is replaced by the ripple structure oriented perpendicular to the laser polarization at the larger fluence of *F* > 45 mJ/cm^2^. At this time, however, the ripple structure formation with the orientation parallel to the laser polarization begins to appear on the laser-exposed surface areas outside the groove during the laser scanning, which indicates a role of energy concentration for the U-shaped groove due to inner-side-wall reflections.

To further verify the significance of the magnetic response of the material for the structure formation with orientation parallel to the laser polarization, we repeated the experiment by replacing the sample material with a foil of Zr_25_Ti_25_Cu_50_ metallic glass, and the adopted laser energy and delay time of double laser pulses were in the range of *F* = 40–70 mJ/cm^2^ and Δ*t* = 0–30 ps, respectively. Being consistent with our theoretical analysis, due to the absence of hysteresis effects, there is no formation of the HSF structures on the sample surface, but only exhibiting the traditional LIPSSs with orientation perpendicular to the laser polarization. More interestingly, the previous studies pointed out that for the material of metallic glass, including the magnetic element of Ni, the HSF structures can be formed upon irradiation of double-pulsed lasers with the delay time less than Δ*t* = 30 ps [44], while only traditional LSF-LIPSSs were observed for the single laser beam irradiation [45,46]. These results dialectically indicate the important roles of both the material hysteresis and magnetization accumulation effects for the anomalous structure formation.

## 4. Conclusions

In summary, we have employed two time-delayed femtosecond laser beams with identical linear polarization to successfully produce intriguing nanostructures inside a groove on the surface of Fe-based metallic glass. At the given time delay of ∆*t* = 5 ps between two sub-pulses, the sole long nanowire formation was observed as the laser polarization was set parallel to the groove direction with the total laser fluence range of *F* = 30–42 mJ/cm^2^, where the measured nanowire width is only about 118 nm, and the spatial length can be continuously extended within a long range. Moreover, at the given total laser fluence of *F* = 30 mJ/cm^2^, the regular structure can be formed within the delay time range of Δ*t* = 20 ps. On the other hand, when the laser polarization became perpendicular to the groove direction, the regular arrays of nanowire structures were developed with orientation perpendicular to the groove direction, where the measured arrangement period was about *Λ* = 192 nm, and the nanowire width approximated 100 nm. Noticeably, in all cases, the generation of a nanowire structure was always spatially oriented parallel to the direction of the laser polarization. Such unique features of nanoscale structures are in sharp contrast to previous reports.

To explore the underlying mechanisms, we have deeply analyzed the physical effects of the SPP wave on the metal surface, which can possess the spin angular momentum to result in the inhomogeneous magnetization of the metal. Especially for the ferromagnetic property of Fe-based metallic glass, the inhomogeneous magnetization can be accumulated during irradiation of double femtosecond laser pulses with picosecond time delays via the hysteresis effect, thus leading to the strong generation of TE-MSW for the spatially periodic ablation of the material. The calculation results matched well with the experimental observations. Moreover, our further experiments demonstrated that for the cases of either double laser irradiation with the time delay larger than Δ*t* = 20 ps, or the single-beam laser irradiation at 1 kHz repetition rate, the formation of such laser-induced nanowire structures became difficult to distinguish, which helps validate our theory.

## Data Availability

Data can be available upon request from the authors.
